# Examining pharmacoepidemiology of antibiotic use and resistance in first-line antibiotics: a self-controlled case series study of *Escherichia coli* in small companion animals

**DOI:** 10.3389/frabi.2024.1321368

**Published:** 2024-02-27

**Authors:** Olivia S. K. Chan, Wendy Wing Tak Lam, Tint Naing, Dorothy Yuen Ting Cheong, Elaine Lee, Ben Cowling, Matthew Low

**Affiliations:** ^1^ Li Ka Shing Faculty of Medicine, School of Public Health, The University of Hong Kong, Pokfulam, Hong Kong SAR, China; ^2^ Soares Avenue Paws and Claws Clinic, Kowloon, Hong Kong SAR, China; ^3^ Sunshine City Veterinary Clinic, Hong Kong, Hong Kong SAR, China; ^4^ The Agriculture, Fisheries and Conservation Department of Hong Kong, Hong Kong, Hong Kong SAR, China; ^5^ Swedish University of Agricultural Sciences, Uppsala, Sweden

**Keywords:** antibiotic use and resistance, pharmacoepidemiology, companion animals, urinary tract infection, self-controlled case series

## Abstract

**Background:**

Clinicians need to prescribe antibiotics in a way that adequately treats infections, while simultaneously limiting the development of antibiotic resistance (ABR). Although there are abundant guidelines on how to best treat infections, there is less understanding of how treatment durations and antibiotic types influence the development of ABR. This study adopts a self-controlled case study (SCCS) method to relate antibiotic exposure time to subsequent changes in resistance patterns. This SCCS approach uses antibiotic exposure as a risk factor, and the development of ABR as an incidence rate ratio (IRR), which can be considered as the multiplicative change in risk for bacteria to become or maintain resistance.

**Aim:**

To investigate the IRR of extensive (more than 7 antibiotic classes), revert, persistent, and directed antibiotic resistance according to the duration and type of antibiotic exposures in *Escherichia coli* (*E. coli*).

**Methods and material:**

We use anonymized veterinary clinical data from dog and cat patients older than 6 months between 2015 and 2020. Patients were considered suitable cases if they received antibiotics and had a minimum of two urinary antibiograms within a 12-month period (the first prior to antibiotics exposure and the second from 1 week to 6 months after exposure). The first antibiogram is conducted before antibiotic exposure (case n=20).

**Findings:**

From 20 individuals and 42 paired antibiograms we found that the IRR = 2 for extensive drug resistance in patients who received short-course antibiotic treatment compared to longer treatments. In contrast, multi-drug resistance IRR = 2.6 for long-course compared to short-course antibiotic treatment. The ratio of *E. coli* isolates that reverted from resistant to sensitive was 5.4 times more likely in patients who received antibiotics for longer than 10 days.

## 1 Introduction

Population and community-based studies demonstrate a general escalation in antibiotic use and subsequent bacterial resistance to antibiotics (Goossens, 2009; Kim et al., 2018). Such studies are crucial in describing the emergence and prevalence of antimicrobial resistance and highlighting trends in where these patterns are strongest and against which antibiotic resistance most commonly occurs. However, it is also important to study antibiotic use and resistance patterns at the clinical and individual levels. Patient-based studies help associate types, doses, and durations of antibiotic use and exposure, plus phenotypically identifiable antibiotic resistance patterns in patients *in vivo* (Metlay, 2002). A clinically oriented antibiotic resistance surveillance network advocates case-based surveillance that can include co-morbidity, antibiotic use history, and whether resistance is reverted or persisted (van Doorn et al., 2020). Such knowledge can inform clinical decision-making and empirical treatment guidelines as well as provide information that can be used for interpreting the results from broader population-based studies. Case-based investigations can provide details such as patients and bacterial profiles, and treatment outcomes based on antibiotics types, duration, resistance persistence, or regression (Ryu et al., 2019).

The Self-Controlled Case Study (SCCS) framework is an analytical approach whereby the relative increase in risk an individual faces after being exposed to an agent is calculated as a multiplicative quantity above a baseline level (Petersen et al., 2016). This approach is a type of Self-Controlled Crossover Observational PharmacoEpidemiology designed to study clinical pharmacology and epidemiology and is usually used to study transient exposures in relation to abrupt outcomes and drug safety (Xilin and Drlica, 2002; Cadarette et al., 2021). It has advantages over other study designs because it uses individuals as their own ‘control’ and, thus, avoids time-invariant confounding factors (Petersen et al., 2016; Hallas et al., 2021). The SCCS design has been applied in the investigation of vaccine safety (Weldeselassie et al., 2011), myocardial infarction and ischemic stroke risk (Katsoularis et al., 2021), and stroke risk after herpes zoster (Katsoularis et al., 2021). In this study, the SCCS framework is extended to examine antibiotic resistance risk in UTIs after exposure to different antibiotics. Here we compare and contrast antibiotic exposure (type and duration of antibiotic use) and antibiotic resistance (ABR) outcome rates between different observation windows of time within the same animal patient so that patients can serve as their own control (Cadarette et al., 2021).

To do this, the study uses antibiotic prescription patterns as the risk agent of exposure, and paired-antibiograms from individual cases to calculate the change in risk (called the Incidence Rate Ratio IRR). The case-based paired antibiogram builds in the time-series exposure consideration by the time intervals and resistant selection window. This approach considers three factors that have not been investigated using such an approach before. *First, Escherichia coli (E. coli*) is the leading pathogen causing urinary tract infections (Erb et al., 2007). Strains of *E. coli* can develop adherence to the urinary tract and cause various forms of urinary tract infection (Källenius et al., 1981). *E. coli* remains a predominant uro-pathogen and an increasingly resistant bacteria in humans (Ronald, 2002), dogs (Thompson et al., 2011), and cats (Hernandez et al., 2014). In the past decade, *E. coli* has increased its resistance to third-generation cephalosporins and harbors more CMY-2-producing enzymes with stronger beta-lactamase capacity (Marques et al., 2017). Second, one should consider methodology to investigate bacterial resistance that may continue or develop after the use of antibiotics (Barbosa and Levy, 2000). Indeed, in addition to the possibility of delayed antibiotic effect, Muiuki et al. describe chromosomal encoding leading to resistance that has shown to persist beyond post-antibiotic utilization, and all resistance to remain “silent” phenotypically (Muriuki et al., 2022). Such time lags in the development of resistance may range from 0-12 months post-antibiotic administration (Poku et al., 2023). Finally, there is the possibility of antibiotic synergy where using a combination of antibiotics, and the concentration dependency of antibiotics (such as doxycycline and erythromycin) may have an effect on antibiotic efficacy and resistance (Chait et al., 2007).

To explore the potential associations between antibiotic resistance and exposure duration, we collected antibiotic use and resistance data from a small animal veterinary clinic in Hong Kong. We use individual animals as cases within the SCCS framework and how their antibiograms change over time in relation to antibiotic use to calculate the IRR risk values. From this, we hope to answer two main questions: First, what are antibiotic resistance patterns for bacteria cultured from UTIs in long- or short-course antibiotic exposure? Second, what are the antibiotic resistance patterns in the absence or presence, of long- or short-first-line amoxicillin-clavulanate, first-generation cephalosporin, and trimethylprim-sulfa use in individual animals?

## 2 Methods and materials

### 2.1 Material

We collected animal patient histories and laboratory records from a veterinary clinic in Hong Kong with five full-time primary care veterinarians. The histories of all patients were validated by comparing their computerized history, pharmaceutical dispensary record, and laboratory test results. Anonymized cases’ identity number, age, and gender of patients, date of consultation, antibiotic prescription by type, dosage and duration, body weight for calculation of dosage, urinary and non-urinary symptoms, urine analysis results, and antibiotic culture and sensitivity (C/S) test results were subsequently recorded for all dog and cat patients who qualified for study case selection (see below). Full history of pharmaceutical use including antibiotics and other than antibiotics, urine analysis finding if any, demographics, and presenting symptoms were recorded.

### 2.2 Study population

The study population is defined as canine and feline patients that were presented or diagnosed with symptoms of urinary tract infection, and subsequent rechecks of these symptoms, between 1 January 2015 and 31 December 2020.

### 2.3 Study case selection

Cases are defined as patients who had more than one urine sample analyzed and laboratory results available as antibiogram records. Cases also had to have full in-house history and antibiotic dispensary records.

### 2.4 Antibiogram inclusion criteria

Antibiograms are included from 1 week to 6 months after one antibiotic use. The criteria are included based on an extrapolation from a study and the clinical observation that 1 week after starting a course of antibiotics to 6 months is when urinary antibiograms are mostly conducted as follow-up to asymptomatic cases or for relapse cases.

In a study of antibiotic resistance emergence in a human intensive care unit, *Pseudomonas aeruginosa* presented no emergence of resistance within 1-3 days of antibiotic use. Significant meropenem resistance emerged within 8 to 15 days with an odds ratio of 14.9 to 421.0 days after antibiotic exposure. In the use of meropenum, antibiotic resistance was associated with the emergence of resistance 8 days after exposure to the antibiotic (Yusuf et al., 2017). In addition, according to veterinary clinical experience locally, follow-up of a urinary tract infection episode is by urine culture for up to about 6 months. A relapse of UTI can also occur in that time span. The intention of a one-week to 6 months’ time window is to capture at least two antibiograms for one particular antibiotic exposure.

### 2.5 Self-controlled case series method

**Figure 1:**
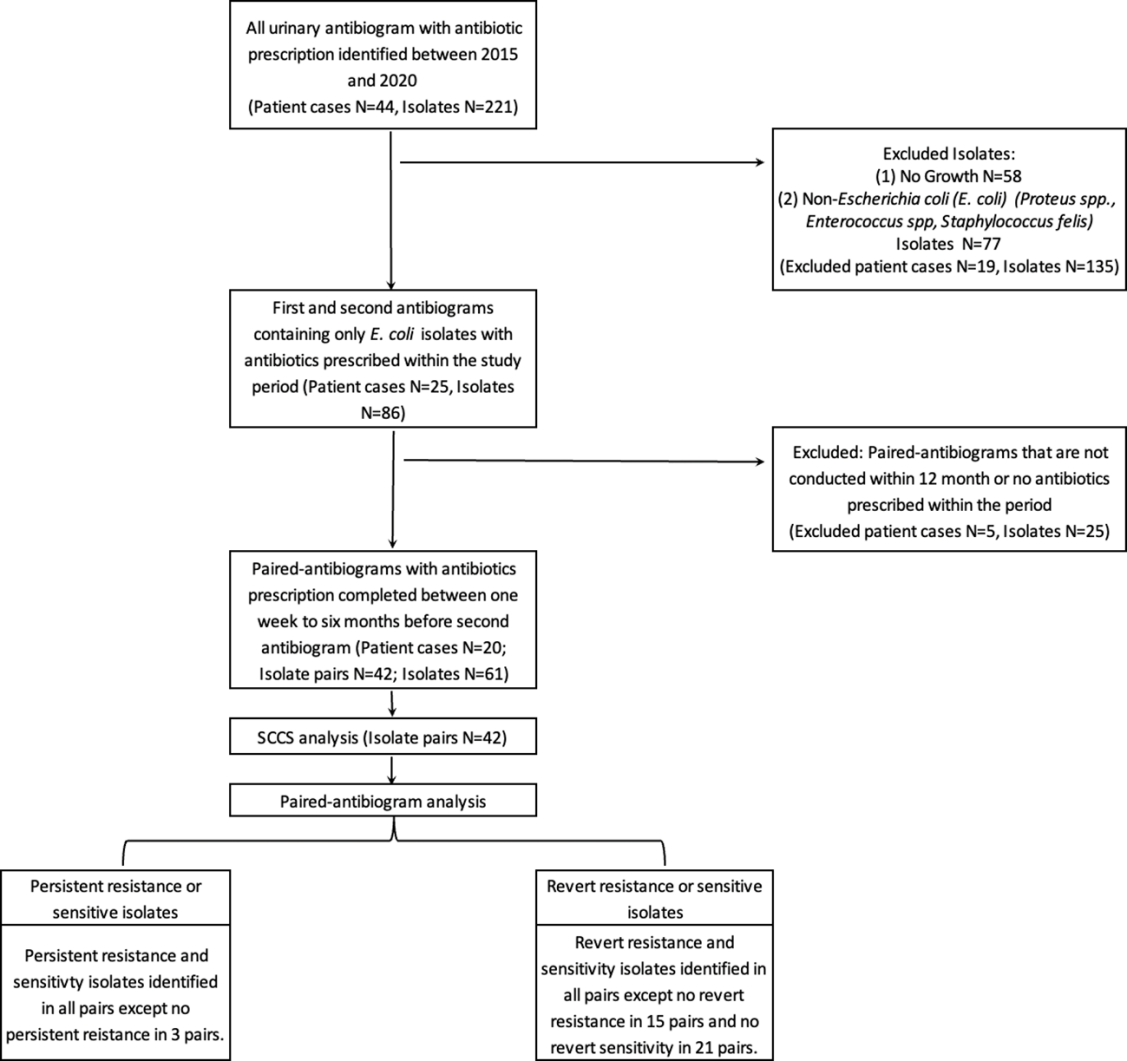
Identification process of antibiotic use and antibiotic resistance cases.

The SCCS method is used here to investigate the association between a patient’s transient exposure to antibiotics and the subsequent detection of *E. coli* antibiotic resistance (**Figure 1**). Thus to be included in this study, cases required at least two antibiogram data points and antibiotic dispensary information. The resistant antibiogram is defined as a “case” and included in the study if the antibiogram could be paired retrospectively with at least one prior antibiogram result conducted within 12 months. Antibiotic exposure that occurred between these two data points was recorded by antibiotic type, use duration, and antibiotic use-to-antibiogram lag time.

### 2.6 Antibiotic exposure duration definitions

Antibiotic exposure time was classified into two categories for this study: (1) ‘long-course’ are cases in which antibiotics were prescribed for more than 10 days, and (2) ‘short-course’ are cases in which antibiotics were prescribed for 10 days or fewer.

### 2.7 Antibiotic resistance definitions

*E. coli* isolates were coded based on their paired antibiograms. Isolates that develop resistance are defined as ‘Revert to Resistant’. Isolates that revert from resistant to sensitive are defined as ‘Revert to Sensitive’. Isolates that do not change their resistance profile between antibiograms remain as ‘Persistent Resistant’ or ‘Persistent Sensitive’.

### 2.8 Analysis

This study uses Incidence Rate Ratios (IRRs) within a Self-Controlled Case Study (SCCS) analytical framework. IRR represents the relative increase in risk an individual faces after being exposed to a risk agent. In ABR, the baseline is sensitive isolates. The incidence rate of resistance examines how antibiotic exposure raises or lowers this ABR incidence. In this study, rather than using a single baseline risk from which IRRs are calculated, we instead calculate IRRs relative to specific ‘baseline’ incidences of antibiotic sensitivity. That is, reversion to resistance is compared to reversion to sensitivity, persistent resistance is compared to persistent sensitivity, extensive-drug resistance is compared to non-extensive resistance, and multi-drug resistance is compared to non-multi-drug resistance.

The incidence rate is defined as the rate of resistance to sensitive paired antibiograms in the 12-month period. The Incidence Rate Ratio (IRR) is the ratio comparing the variables of interest, for instance long- and short-antibiotic course and, presence or absence of an antibiotic. The incidence rate ratio equals to incidence rate of resistance studied to long-course antibiotics divided by the Incidence rate of resistance to short-course antibiotics. The 95% confidence interval is *p*ˆ ± 1.96 √ (*p*ˆ (1- *p*ˆ)/n). Thus the IRRs reported in this study represent a calculation of the multiplicative increases in incidence in ABR that patients experience given their antibiotic exposure and previous sensitivity or resistance history of the bacteria in their urinary tract. The IRRs are all based on paired antibiograms because doing so provides clarity on the reversion or persistence of resistance. The resistance or sensitivity paired-antibiograms are stratified by antibiotic exposure duration.

IRRs are compared in two main scenarios. First, IRRs of resistance variation in long- or short-course antibiotic exposure. Resistance is studied in: (a) Reversion to resistance compared to sensitivity, which is when isolates change phenotype; (b) Persistent resistance, which is when isolates remain resistant throughout the duration of observation; (c) Extensive-drug resistance, which is when isolates are resistant to 7 or more classes of antibiotics; and (d) Multi-drug resistance, which is when isolates are resistant to more than 3 classes of antibiotics. Second, IRRs of resistance patterns are studied according to absence, presence, and long- and short-course of first-line antibiotics uses; namely amoxicillin-clavulanate, first-generation cephalosporin, and trimethylprim-sulfa.

## 3 Results

### 3.1 Characteristics of the data

**Figure 2:**
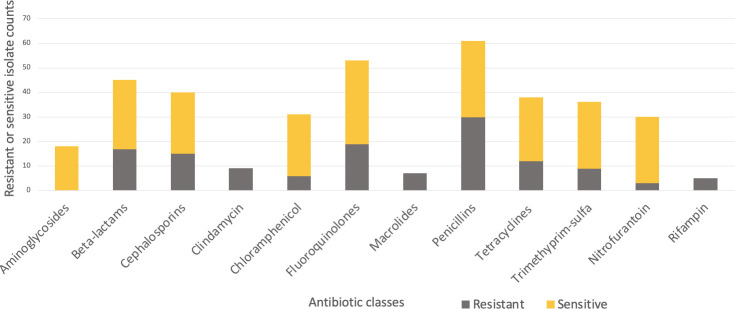
Resistant (grey-bottom) and sensitive (yellow-top) isolate counts summarized by antibiotic classes.

A total of 44 patients and 221 urinary antibiograms were identified. These patients were presented with urinary symptoms and were prescribed at least one course of antibiotics between 2015 to 2020. The most commonly isolated pathogen was *Escherichia coli* (*E. coli*) (39%; N=86) (**Figure 1**). This study focuses on *E. coli* and a total of 20 cases and 42 pairs of antibiograms were included in the study. These cases came from 16 canine and 4 feline patients ranging from 1 to 17 years old. Cases included seven neutered female canines and two felines, seven neutered male canines and two felines, and two non-neutered male canine patients. Ten classes of antibiotics were prescribed to the patients including aminoglycosides, beta-lactams, cephalosporins, fluoroquinolones, macrolides, penicillins, tetracyclines, trimethyprim-sulfa, nitroimidazole (metronidazole), and the urinary anti-infective nitrofurantoin. Resistance and sensitivity counts to these antibiotics are shown in **Figure 2**. In most cases, these antimicrobials were prescribed for urinary tract infections, but some were also prescribed for other conditions. *E. coli* isolates indicate resistance to rifampin but no prescription is identified.

### 3.2 Long- and short-course antibiotic exposure, antibiotic sensitivity, and resistance

There are 15 paired antibiograms that indicate reversion from sensitive to resistant and 11 paired antibiograms that revert from resistant to sensitive. The incidence rate (IR) of pairs that revert to resistance is 0.7 (IR=7/10; N=17) subsequent to long-antibiotic exposure. The IR of pairs that revert to resistance is 8 (IR=8/1; N=9) subsequent to short-antibiotic exposure. The IRR of paired-antibiograms that revert to resistance is 0.09 (IRR=0.7/8; N=26) comparing long- to short-course antibiotic treatment. The incidence of revert resistance is lower in long-course antibiotic treatment compared to short-course antibiotic treatment (**Table 1**).

**Table 1:** Paired-antibiogram counts per 12 months, incidence rate and rate ratio of revert resistance, revert sensitivity, persistent sensitivity, extensive-drug resistance, and multi-drug resistance comparing long- and short-course antibiotic treatment.

	Paired-antibiograms (Counts)/12 months			
	Revert to Resistant	Revert to Sensitive	Incidence rate	Incidence rate ratio
**Long-course antibiotic treatment**	7	10	0.7	0.09 (CI: 0.05, 0.2)
**Short-course antibiotic treatment**	8	1	8	
	Persist Resistance	Persist Sensitivity	Incidence rate	Incidence rate ratio
**Long-course antibiotic treatment**	4	14	0.28	1.3 (CI: 0.4-4.3)
**Short-course antibiotic treatment**	2	9	0.22	
	Extensive-drug resistance (EDR)	Not EDR	Incidence rate	Incidence rate ratio
**Long-course antibiotic treatment**	2	22	0.1	0.5 (CI: 0.08, 3.0)
**Short-course antibiotic treatment**	2	11	0.2	
	Multi-drug resistance (MDR)	Not MDR	Incidence rate	Incidence rate ratio
**Long-course antibiotic treatment**	16	3	5.3	2.65 (CI: 1.2, 5.9)
**Short-course antibiotic treatment**	10	5	2	

There are six paired antibiograms that are persistently resistant to antibiotics. The IR of isolates with persistent resistance is 0.28 (IR=4/14; N=18) subsequent to long-antibiotic exposure. The IR to persistent resistance is 0.22 (IR=2/9; N=11) subsequent to short-antibiotic exposure. The IRR of persistent resistance is 1.3 in long-antibiotic courses compared to short (Ratio=0.28/0.22; N=29) (**Table 1**). The incidence of persistent resistance is higher with long-course antibiotic treatment than short-course antibiotic treatment.

Two additional IRRs measure extensive- and multi-drug resistance. There are four paired antibiograms that indicate extensive antibiotic resistance with resistance to 7 or more classes of antibiotics. The IR of extensive-drug resistance is 0.1 (IR=2/22; N=24) subsequent to long-antibiotic exposure. The IR of extensive resistance is 0.2 (IR=2/11; N=13) subsequent to short-antibiotic exposure. The IRR for extensive-drug resistance is two times higher (IRR=0.2/0.1; N = 37) in pairs that are exposed to antibiotics for 10 days or shorter (**Table 1**). The isolates were resistant to beta-lactams, cephalosporin, chloramphenicol, fluoroquinolone, nitrofurantoin, penicillin, tetracycline, and trimethylprim-sulfa. One patient with extensive antibiotic resistance was prescribed long-course amoxicillin-clavulanate and a second patient with long course nitrofurantoin. Both patients diagnosed with extensive antibiotic resistance who were on short-course antibiotics were prescribed cephalexin. The incidence of extensive-drug resistance is lower with long-course antibiotic treatment than short-course antibiotic treatment.

There are 26 paired antibiograms that indicate multi-drug resistance. The IR of multi-drug resistance is 5.3 (IR=16/3; N=22) subsequent to long-antibiotic exposure. The IR of multi-drug resistance is 2.0 (IR=10/5; N=10) subsequent to short-antibiotic exposure. The IRR of multi-drug antibiotic resistance is 2.7 (IRR=5.3/2.0; N = 37) in isolate pairs exposed to antibiotics for longer than 10 days (**Table 1**). These patients receive amoxicillin-clavulanate, cephalosporins, enrofloxacin, tetracycline, nitrofurantoin, and trimethylprim-sulfa and are resistant to classes 4 to 6 among these antibiotics. The incidence of multi-drug resistance is higher with long-course antibiotic treatment than short-course antibiotic treatment.

### 3.3 Resistance in amoxicillin-clavulanate, cephalosporin, and trimethylprim-sulfa exposure

Amoxicillin-clavulanate, cephalosporin, and trimethylprim-sulfa are recommended first-line antibiotics in the treatment of urinary tract infections in feline and canine patients. Directed resistance and sensitivity from long- and short-antibiotic use are summarized in **Table 2**.

**Table 2:** Paired-antibiogram counts per 12 months, incidence rate, and rate ratio of resistance and sensitivity cases exposed to first-line antibiotics.

	Paired-antibiograms (Counts)/12 months			
	Resistance to Amoxicillin-Clavulanate	Sensitive to Amoxicillin-Clavulanate	Incidence rate	Incidence rate ratio
**Use Amoxiclav**	5	10	0.5	0.25 (CI: 0.03, 1.8)
**No Amoxiclav use**	12	6	2	
	Resistance to first- generation Cephalosporin	Sensitive to first- generation Cephalosporin	Incidence rate	Incidence rate ratio
**Use Cephalosporin**	2	6	0.3	0.5 (CI: 0.14, 1.9)
**No Cephalosporin use**	7	12	0.6	
	Resistance to Trimethylprim-sulfa	Sensitive to Trimethylprim-sulfa	Incidence rate	Incidence rate ratio
**Use Trimethylprim-sulfa**	1	1	1	1.4 (CI: 0.3, 5.5)
**No Trimethylprim-sulfa use**	12	18	0.7	

There are 17 paired-antibiogram resistant and 16 paired-antibiogram sensitive to amoxicillin-clavulanate. The IRs of resistant paired isolates is 0.5 (IR=5/10; N=15) subsequent to amoxicillin-clavulanate use and 2 (IR=12/6; N=18) subsequent to not using amoxicillin-clavulanate. The IRR of directed resistance to amoxicillin-clavulanate is 0.25 (IRR=0.5/2; N=33) (**Table 2**). The incidence of directed resistance is lower using amoxicillin-clavulanate treatment than not.

There are 9 paired-antibiogram resistant and 18 paired-antibiogram sensitive to first-generation cephalosporin. The IRs of resistant paired-isolates is 0.3 (IR=2/6; N=8) subsequent to first-generation cephalosporin use and 0.6 (IR=7/12; N=19) subsequent to no first-generation cephalosporin use. The IRR of directed resistance to first-generation cephalosporin is 0.5 (IRR=0.3/0.6; N=27) (**Table 2**). The incidence of directed resistance is lower using first-generation cephalosporin treatment than not.

There are 13 paired-antibiogram resistant and 19 paired-antibiogram sensitive to trimethyprim-sulfa. The IRs of resistant paired isolates is 1 (IR=1/1; N=2) subsequent to trimethyprim-sulfa use and 0.7 (IR=12/18; N=30) subsequent to not using trimethylprim-sulfa. The IRR of directed resistance to trimethylprim-sulfa is 1.4 (IRR=1/0.7; N=32) (**Table 2**). The incidence of directed resistance to trimethylprim-sulfa is higher using trimethylprim-sulfa treatment than not.

### 3.4 Long- and short-antibiotic use and change of resistance and sensitivity in first-line antibiotics

The pattern of change to resistance and sensitivity is investigated in two first-line antibiotics. The reversion to resistance meant isolates changed from sensitive to resistant phenotype.

There are 11 paired antibiograms that reverted resistance and 6 pairs that reverted sensitivity to amoxicillin-clavulanate. The IR of reversion to resistance is 2 (IR=8/4; N=12)) subsequent to long-course amoxicillin-clavulanate use and 1.5 (IR=3/2; N=5) subsequent to short-course amoxicillin-clavulanate use. The IRR is 1.3 (IRR=2/1.5, N=17) (**Table 3**). The incidence of reverted resistance is higher with using long-course amoxicillin-clavulanate treatment than with short-course amoxicillin-clavulanate treatment.

**Table 3:** Paired-antibiogram counts per 12 months, incidence rate and rate ratio of revert resistance and sensitivity in cases comparing long-course and short-course of two first-line antibiotics.

	Paired-antibiograms (Counts)/12 months			
	Revert resistance to Amoxicillin-Clavulanate	Revert sensitivity to Amoxicillin-Clavulanate	Incidence rate	Incidence rate ratio
**Long-course Amoxicillin-clavulanate treatment**	8	4	2	1.3 (CI: 0.58, 2.9)
**Short-course Amoxicillin-clavulanate treatment**	3	2	1.5	
	Revert resistance to first-generation Cephalosporin	Revert sensitivity to first-generation Cephalosporin	Incidence rate	Incidence rate ratio
**Long-course first-generation Cephalosporin**	1	2	0.5	0.1 (CI: 0.02, 0.5)
**Short-course first-generation Cephalosporin**	5	1	5	

There are six paired antibiograms that reverted resistance and three pairs that reverted sensitivity to first-generation cephalosporin. The IRs of reversion to resistance are 0.5 (IR=1/2; N=3) subsequent to long-course amoxicillin-clavulanate use and 5 (IR=5/1; N=6) subsequent to short-course amoxicillin-clavulanate use. The IRR is 0.1 (IRR=0.5/5, N=9) (**Table 3**). The incidence of reverted resistance is lower with using long-course first-generation cephalosporin treatment than with short-course first-generation cephalosporin treatment.

### 3.5 Period between antibiotic use and the second antibiogram

The period between antibiotic exposure to the second antibiogram is defined as temporally proximal or distal exposure. Proximal exposure is defined as the period within 7 days between the antibiotic used and when the second antibiogram is conducted. Distal exposure is defined as the period between 8 days and 6 months when an antibiotic is used and when the second antibiogram is conducted. Per this categorization of data, there are 5 paired-antibiograms from doxycycline use, 6 from cephalosporin use, 10 from amoxicillin-clavulanic acid use, 2 from nitrofurantoin use, and 1 from fluoroquinolone use (**Table 4**). Of the 5 pairs of antibiograms with doxycycline exposure, 3 pairs are defined as distal exposure and each antibiogram demonstrated either reverted or persistent resistance to fluoroquinolone; 2 of 5 pairs are defined as proximal exposure with persistent sensitivity to fluoroquinolone. Of the 6 pairs of antibiograms exposed to cephalosporin, 3 are exposed distally and 3 proximally. Of the 3 antibiograms with exposure to cephalexin distally, 2 reverted to resistance and 1 remained sensitive to cephalosporin. Of the 3 antibiograms with exposure to cephalexin proximally, 2 remained sensitive and 1 reverted to resistance to cephalosporin. Of the 10 pairs of antibiograms with exposure to amoxicillin-clavulanic acid, 7 were exposed distally and 3 were exposed proximally. Of these 7 pairs of antibiograms with exposure to amoxicillin-clavulanic acid distally, 3 remained sensitive to amoxicillin-clavulanic acid and 4 reverted or persisted resistance. Of the 3 pairs of antibiograms that were exposed to amoxicillin-clavulanic acid proximally, 2 pairs remained sensitive and 1 reverted to resistance to amoxicillin-clavulanic acid. Of the 2 pairs of antibiograms that were exposed to nitrofurantoin, one pair was from distal exposure and the other proximal. There is revert resistance to nitrofurantoin from the distal exposure and persistent sensitivity to proximal exposure. The paired-antibiogram with marbofloxacin proximal exposure demonstrated persistent sensitivity to marbofloxacin. Details of antibiograms are listed in **Table 4**.

**Table 4:** The time span, antibiotic exposure, and the second antibiogram.

		Paired-antibiogram sensitivity and resistance			
Antibiotic class	Time of exposure to antibiogram	Revert resistance	Persistent resistance	Revert sensitivity	Persistent sensitivity
Tetracycline					
Doxycycline	DE, 2 months	Doxycycline			
Doxycycline	DE, 2 months		Doxycycline		
Doxycycline	DE, 1 month	Doxycycline			
Doxycycline	PE, 4 days				Doxycycline
Doxycycline	PE, 3 days				Doxycycline
Cephalosporin					
Cephalexin	DE, 1 month	Cefazolin			Cefixime, Cefovecin, Cefoxitin, Cefpodoxime, Ceftazidime, Ceftriaxone, Cefuroxime, Cephalexin
Cephalexin	DE, 2 months	Cefovecin, Ceftriaxone			Cefpodoxime, Cephalexin
Cephalexin	DE, 2 months				Cefovecin, Cefpodoxime, Ceftriaxone, Cephalexin
Cephalexin	PE, 3 days				Cefovecin, Cefpodoxime, Ceftibuten, Ceftriaxone, Cephalothin
Cephalexin	PE, 7 days				Cefovecin, Ceftriaxone
Cephalexin	PE, 3 days	Cefovecin, Cefpodoxime, Cephalexin			Ceftriaxone
Amoxicillin-clavulanic acid					
Amoxicillin-clavulanic acid	DE, 2 months				Amoxicillin-clavulanic acid
Amoxicillin-clavulanic acid	DE, 5 months		Amoxicillin-clavulanic acid		
Amoxicillin-clavulanic acid	DE, 2 months				Amoxicillin-clavulanic acid
Amoxicillin-clavulanic acid	DE, 3 months	Amoxicillin-clavulanic acid			
Amoxicillin-clavulanic acid	DE, 1 month	Amoxicillin-clavulanic acid			
Amoxicillin-clavulanic acid	DE, 4 months	Amoxicillin-clavulanic acid			
Amoxicillin-clavulanic acid	DE, 5 months				Amoxicillin-clavulanic acid
Amoxicillin-clavulanic acid	PE, 6 days				Amoxicillin-clavulanic acid
Amoxicillin-clavulanic acid	PE, 4 days				Amoxicillin-clavulanic acid
Amoxicillin-clavulanic acid	PE, 3 days	Amoxicillin-clavulanic acid			
Nitrofurantoin					
Nitrofurantoin	DE, 2 months	Nitrofurantoin			
Nitrofurantoin	PE, 10 days				Nitrofurantoin
Fluoroquinolone					
Marbofloxacin	PE, 10 days				Marbofloxacin

DE, Distal exposure; PE, Proximal exposure.

## 4 Discussion

Lopatkin et al. described that sensitive bacteria can displace resistant counterparts if resistant genes are costly (Lopatkin et al., 2017). However, the authors also demonstrate resistant conjugal plasmids that are transferred at high rates in *Escherichia coli (E. coli)* even when costly (Lopatkin et al., 2017). In another study by Palomino et al, the authors demonstrated metabolic genes that can impact antibiotic resistance genes and antibiotic susceptibility measured by MIC (Palomino et al., 2023). Some of these mechanisms of resistance may lead to variation in resistance patterns observed clinically. We ask whether antibiotic use duration variation leads to phenotypic resistance persistence or reversion observed in clinical cases. In this study, we wish to identify if there is a plausible pattern of antibiotic use duration and resistance persistence or reversion. This epidemiology study intends to describe resistant or sensitive phenotypic traits stratified by clinical antibiotic use duration.

According to the IRR and MDR between short- and long-course antibiotic administration (**Table 2**), long-course antibiotic treatment is possible to be inside the boundary of mutant prevention concentration according to the concept of mutant prevention concentration (Smith et al., 2003). Short-course antibiotic treatment may have enriched mutant fraction and therefore is resistant mutant-prone and leads to resistance more rapidly. Furthermore, it is clinically relevant to investigate from a short-course antibiotic treatment perspective. That is, a few questions can be further postulated in case IRRs are less than one in some resistance circumstances in short-course antibiotic use. For instance, whether short-course amoxicillin-clavulanate can reduce revert resistance directed to its use and how to use amoxicillin-clavulanate and first-generation cephalosporin in a way that can reduce directed resistance.

This study adopts a case-crossover method within the family of self-controlled study designs (Lewer et al., 2022). This study introduces the approach of pairing antibiograms that include temporal changes in IRR investigation. The pairing can be a promising methodology that should be further explored in resistance prevalence studies because it preserves the comparison framework for case- and control window of antibiotic use and its resistance within a patient and therefore limits inter-patient and inter-microbial confounders. One strength that pertains to this study methodology is to follow variations of events in a patient over time. It includes the reversion and persistence of antimicrobial sensitivity and resistance at the patient level. Another strength of this study design is to minimize any patient-to-patient confounding factors. The study method to analyze data as paired data is also conservative in that it is not a point prevalence investigation but a revert resistance or persistent resistance to ensure there are observations on change of bacteria’s phenotypic expression based on antibiotic exposure. Because of SCCS, temporal variation of baseline incidence can be allowed. Another strength of adopting the SCCS method in the investigation of antibiotic use and resistance is it automatically controls for multiplicative time-invariant confounders, even when these are unmeasured or unknown. This method is most frequently used to study the safety of vaccines and pharmaceutical drugs (Farrington et al., 2018).

This study presents several opportunities to study polypharmacy that deserve further exploration in a study with larger sample sizes. In addition to the combined use of antibiotics, the effects of steroids, non-steroidal anti-inflammatory drugs (NSAID), antacids, and immunomodulatory medications are often administered with antibiotics. In one study, nitrofurantoin resistance is negatively associated with amoxicillin resistance because of the “high cost in nitrofurantoin resistance” (Pouwels et al., 2018; Pouwels et al., 2019). This may be further explored with our finding that nitrofurantoin resistance is associated with trimethoprim resistance. The findings from this study do not provide sufficient evidence to compare with the description by Pouwels et al. (2019). There is a mix of fluoroquinolone, cephalosporin and trimethoprim-sulfate resistance to enrofloxacin, marbofloxacin, and pradofloxacin-use and no pattern can be interpreted (**Supplementary Figure 1**). Co-selection or by-stander selection, defined as inadvertent pressure imposed by antibiotic treatment on microbes other than targeted pathogen, has been hypothesized as a factor that propagates antibiotic resistance. There is still contention about whether bystander selection is the/a rule rather than the exception (Tedijanto et al., 2018). Among non-antimicrobial drugs, Pilotto et al. describe that proton pump inhibitors potentiate risk in antibiotic resistance (Pilotto et al., 2000). In addition, Verma et al. propose NSAID may induce antibiotic resistance (Verma et al., 2018). It is important to study clinical antibiotic resistance that changes over time, co-morbidities, and medications such as NSAIDS, steroids, and antacids that were on board, and durations of prescription that can optimize antibiotic prescription. In cases of larger sample size, one can investigate antibiotic resistance stratified by specific antibiotics or non-antibiotic polypharmacy.

There are a number of limitations to this study design. Case-based surveillance is a labor-intensive way to curate clinical information. The pairing of antibiograms is time-consuming. This study also contains a small sample size and is thus susceptible to sampling biases and validity concerns. There are also a number of problems with using this methodology to study antibiotic use and resistance. First, the association between antibiotic use may or may not be immediate. That is, there is a latency period for the development of a disease or condition. Second, the phenotypic resistance pattern may not be statistically clear with antibiotic use. Third, there are possibilities of unknown effects such as carryover and period effects of antibiotic use and resistance. This is because the effect of one exposure period may overlap with another period of exposure. Fourth, the limitation of *in vivo* retrospective study is the lack of control of antibiotic use, but the nature of antibiotic use and resistance as a function of time provides accounts for the possible clinical output that exists within patients, between patients, and different classes of antibiotics. The limitation of using the SCCS method also applies to the data denominator in a catchment area of clinics, which may or may not represent not uniform antibiotic use (Whitaker et al., 2009). The challenge to using this method, compared to vaccination and meningitis studies (Douglas et al., 2009; Farrington et al., 2018), is when the event times, antibiotic resistance, and exposure times (antibiotic use) are non-uniform, as opposed to vaccination, in which exposure times are fixed. As mentioned by Whitaker, the case series method incorporates a feature that controls for age, fixed confounders (Maclure and Mittleman, 2000), and for multiple events (Whitaker et al., 2006).

In addition, *P. aeruginosa* resistance emergence serves as a reference for antibiogram inclusion criteria but the major limitation is that *P. aeruginosa* can emerge resistance differently from *E. coli.* Indeed, authors do not know the timeline of the mutations, post-translational, and other mechanisms that can attenuate or do not attenuate antibiotic activities. It is this unknown that inspires this epidemiological investigation. The intention of a one-week to 6 months time window is to capture at least two antibiograms for one particular antibiotic exposure. The choice of study window is based mainly on extrapolation of the emergence of resistance in a hospital study and clinical availability of antibiograms between 1 week to 6 months post-antibiotic exposure. This window poses a limitation for changes that occur after 6 months. This limitation can lead to an incomplete capture of the resistant pattern. In further studies, the window can be extended.

Further study on the application of SCCS in antibiotic resistance studies will help understand the cumulative effect of the occurrence of antibiotic use-resistance combination and add to the body of use and resistance patterns. For instance, there are baseline data in human medicine that would be useful to document in veterinary medicine. A meta-analysis study generated a significant pooled odds ratio of 2.3 (95% confidence interval 2.2 to 2.5). This study describes knowledge that in human studies, *E. coli* is resistant to quinolone (17%), beta-lactam (14%), and sulphonamide (13%). It is also described that the time between consumption and resistance is about six months or shorter (53%) or more than six months (23%) in different cases. These are data from cross-sectional and ecological studies in different countries (Bell et al., 2014). In further studies, it will be good to expand the database on the number of samples as well as the time between consumption and resistance. This can help investigate the relative incidence specific to antibiotic use class, time, and resistance. As described by Cherny et al., further investigation should also study whether the occurrence of one antibiotic resistance may be associated with an increased or decreased probability of subsequent resistances (Cherny et al., 2020).

There was data collected on the period and changes between antibiotic use and the second antibiogram. However, a larger sample size is needed. There are insufficient cases in each antibiotic group to conclude between antibiotic use and the reversion or persistence of resistance or sensitivity in the antibiogram. There is also a varying distribution of resistance and sensitivity according to the period between antibiotic use and the second antibiogram. However, the time-span variable should be included in the model as a confounder to be investigated with a larger dataset.

The study is prompted by the variations of persistent and reversive resistance that are observed in clinical patients. There is resistance observed in UTI in *E. coli* in clinical cases weeks after the antibiotic course is completed. On the other hand, some cases revert from resistance to sensitivity. Holm et al. studied antibiotic courses and defined short courses as fewer than or equal to 5 days and long courses as longer than or equal to 7 days in patients with laryngitis (Holm et al., 2020). Kyriakidou et al. conducted a meta-analysis on antibiotic use and described 7 to 14 days as a short course and 14 to 42 days as a long course in patients with pyelonephritis (Kyriakidou et al., 2008). Both studies focused on treatment success, relapse of infection, and adverse reactions. A study by Spellberg and Rice described how each day of antibiotic use confers decreased additional benefits to clinical cure while increasing the burden of harm in the form of antibiotic resistance (Spellberg and Rice, 2019). It was further described in a systematic review, the duration of antibiotic therapy for community-acquired pneumonia between 3 to 14 days does not change the outcome of antibiotic treatment. In terms of antibiotic resistance, the author described randomized control studies that demonstrated shorter courses decreased resistance in respiratory secretions in human. Further study on difference in terms of cumulative changes of phenotypic resistance per antibiotic use will be useful.

The study intends to describe the variation in clinical and phenotypic observation and substantiate good practice with antibiotic use duration recommendations. In a study by Marque et al, the molecular changes that caused resistance also appear to occur after instances of antibiotic presence (Marques et al., 2017). Author Munoz-Price proposed alteration of the gut microbiome is a possible influence and therefore causes time-dependent exposure instead of time-fixed exposure which truncates antibiotic resistance hazard on the day of exposure (Munoz-Price et al., 2016). For instance, “return to susceptibility was observed over time…” and in a case, the author described a canine patient resistant to ampicillin, tetracycline, TMS, cephalosporin, kanamycin, and chloramphenicol who only received ampicillin (Marques et al., 2017). There is also potential for the study of the antibiotic influence on resistance that extends beyond the antibiotic’s half-life of elimination. The next question can include when antibiotic use has been the same, and genomic studies can help understand the molecular changes.

## 5 Conclusion

The pattern of resistance incidence of long-course and short-course antibiotic treatment was inconsistent. IRRs of revert-to-resistant and extensive-drug resistance were less than one, which suggests fewer such incidences occur in long-course antibiotic treatment. In contrast, IRRs of persistent resistance and multi-drug resistance were larger than one which indicates both persistent and multi-drug resistance incidence are more frequent in long-course antibiotic treatment. It is clinically relevant to further investigate whether resistance reversion and extensive-drug resistance can be lowered with long-course antibiotic treatment. This observation also ties in with fewer reverted resistance incidences directed towards first-generation cephalosporin in long-course first-generation cephalosporin use.

## Data availability statement

The original contributions presented in the study are included in the article/**Supplementary Material**. Further inquiries can be directed to the corresponding author.

## Ethics statement

The manuscript presents research on data. Ethical approval was obtained under HKU/HA HKW IRB Reference number UW 18-206.

## Author contributions

OC: Conceptualization, Data curation, Formal analysis, Funding acquisition, Investigation, Methodology, Writing – original draft, Software, Visualization. WL: Project administration, Supervision, Writing – review & editing. TN: Data curation, Investigation, Project administration, Writing – review & editing. DC: Data curation, Project administration, Resources, Writing – review & editing. EL: Project administration, Resources, Supervision, Writing – review & editing. BC: Conceptualization, Investigation, Methodology, Supervision, Writing – review & editing. ML: Formal analysis, Methodology, Validation, Writing – review & editing.
