# Supplementary material for: Examining pharmacoepidemiology of antibiotic use and resistance in first-line antibiotics: a self-controlled case series study of Escherichia coli in small companion animals
Source: Front Antibiot. 2024 Feb 27;3:1321368. doi: 10.3389/frabi.2024.1321368 (PMC11731916; doi:10.3389/frabi.2024.1321368)
Supplement: Supplementary file 1 [file Image_1.pdf]

Supplementary Figure 1. An example of temporal pattern of antibiotic use and resistance. Fluoroquinolone use and resistance profile of *E. coli*.

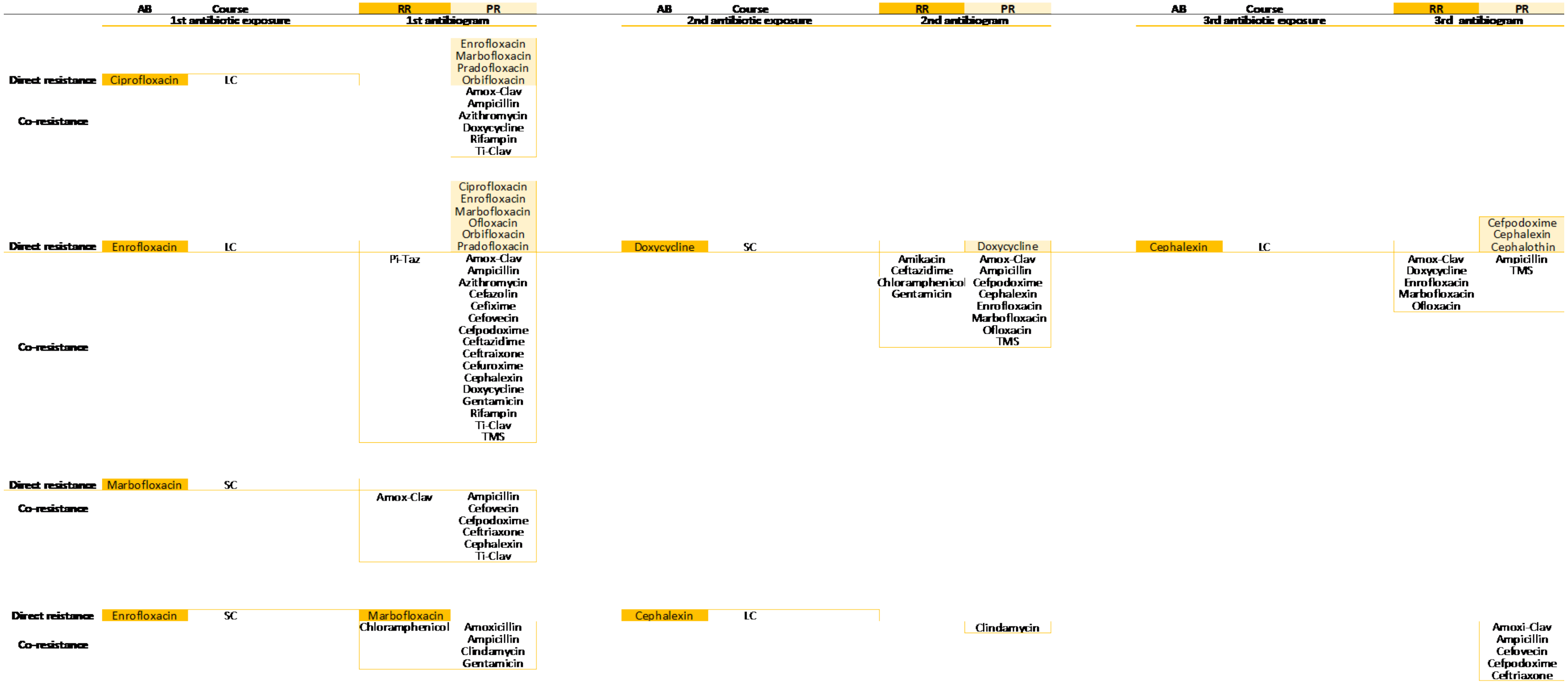

Keys: TMS = Trimethoprim-sulfur, Amox-Clav = Amoxicillin-clavulanate, Ti-Clav = Ticarcillin-clavulanate, Pi-Taz = Piperacillin-clavulanate SC = Short course (=<10 days), LC = long course (>10 days), RR = revert resistance, PR = persistent resistance
